# A Review on Potential Mechanisms of* Terminalia chebula* in Alzheimer's Disease

**DOI:** 10.1155/2016/8964849

**Published:** 2016-01-28

**Authors:** Amir R. Afshari, Hamid R. Sadeghnia, Hamid Mollazadeh

**Affiliations:** ^1^Pharmacological Research Center of Medicinal Plants, School of Medicine, Mashhad University of Medical Sciences, Mashhad 917794-8564, Iran; ^2^Neurocognitive Research Center, School of Medicine, Mashhad University of Medical Sciences, Mashhad 917794-8564, Iran

## Abstract

The current management of Alzheimer's disease (AD) focuses on acetylcholinesterase inhibitors (AChEIs) and NMDA receptor antagonists, although outcomes are not completely favorable. Hence, novel agents found in herbal plants are gaining attention as possible therapeutic alternatives. The* Terminalia chebula* (Family: Combretaceae) is a medicinal plant with a wide spectrum of medicinal properties and is reported to contain various biochemicals such as hydrolysable tannins, phenolic compounds, and flavonoids, so it may prove to be a good therapeutic alternative. In this research, we reviewed published scientific literature found in various databases: PubMed, Science Direct, Scopus, Web of Science, Scirus, and Google Scholar, with the keywords:* T. chebula*, AD, neuroprotection, medicinal plant, antioxidant, ellagitannin, gallotannin, gallic acid, chebulagic acid, and chebulinic acid. This review shows that* T. chebula* extracts and its constituents have AChEI and antioxidant and anti-inflammatory effects, all of which are currently relevant to the treatment of Alzheimer's disease.

## 1. Introduction

Alzheimer's disease (AD) is the leading cause of neurodegenerative disease in the geriatric population, accounting for approximately 5 million cases of dementia in the USA according to estimates from the Alzheimer's Association in 2015. Since the disease incidence increases with the progression of age, the risk of developing AD doubles every 5 years, beginning at age 65. Given the growing elderly population in developed countries, projections of future AD prevalence show a fourfold increase through 2050. Consequently, AD has become a major economic health burden because of accrued high healthcare costs, morbidity, and mortality, not to mention the financial burden it has on family members and caregivers due to lost wages and productivity [1].

The pathogenesis of AD is very complicated, but typically cerebral atrophy is clearly evident in postmortem and imaging studies. Combined cortical, limbic, and subcortical pathology leads to dementia typical of AD. Classic microscopic features include neurofibrillary tangles and senile plaques. Also, the clinical features of AD are commonly characterized by psychopathological signs such as language deterioration, memory loss, visuospatial impairment, and poor judgment [2]. Additionally, in AD there is a gradual loss of various neurotransmitters particularly in the basal forebrain. Cholinergic transmission is the earliest and most conspicuously affected in AD. The nucleus basalis in the basal forebrain is affected comparatively early in the process and acetylcholine levels inside the spinal fluid and brain of AD patients quickly reduce with the progression of the disease. This fact supports the cholinergic hypothesis that acetylcholine diminution results in the cognitive decline observed in AD patients finally go to the first symptomatic treatment of AD [3].

Most of the drugs presently available to treat AD are acetylcholinesterase inhibitors (AChEIs): tacrine [4], rivastigmine [5], donepezil [6], and galantamine [7]; but unfortunately all of these drugs have limited effectiveness and side effects [4]. On the other hand, medicinal plants are starting to take an important role in disease treatment, in particular to treat psychiatric and neurological disorders. One reason is because of the discontentment with conventional treatments and another is because patients are seeking greater self-control over their healthcare decisions [8].

It seems that due to the indiscriminate and excessive use of drugs, their costs, side effects, and interactions, herbal medicines can be a suitable alternative to treat diseases because of their low costs, availability, and fewer drug interactions [9]. Therefore, the search for a new pharmacotherapy from medicinal plants to treat neurodegenerative disorders has remarkably advanced and there are several studies and documents that indicate a significant role of herbal medicines in the treatment of AD [10–12]. One particular herbal remedy is* Terminalia chebula *Retz. (Combretaceae) because of its numerous and different types of phytoconstituents such as polyphenols, terpenes, anthocyanins, flavonoids, alkaloids, and glycosides. In traditional medicine, the fruits of the* T. chebula*, which hold various chemically active compounds responsible for its medicinal properties, have been used in Unani, Ayurveda, and homeopathic medicine since antiquity to treat geriatric diseases and improve memory and brain function [13, 14]. It is also commonly used to treat numerous diseases such as cancer, cardiovascular diseases, paralysis, leprosy, ulcers, gout, arthritis, epilepsy, cough, fever, diarrhea, gastroenteritis, skin disorders, urinary tract infection, and wound infections [15, 16]. Recent studies show that* T. chebula *is effective in the treatment of diabetes [17], bacterial and fungal infections [18, 19], immunodeficiency diseases [20, 21], hyperlipidemia [22], liver diseases [15, 23], stomach ulcer [24], and wounds [25]. Other pharmacological properties and beneficial effects of* T. chebula* are summarized in Table 1.

Hence, this review article aims to sum up the published literature on the pharmacology and phytochemistry of* T. chebula* and its effects on the progression and treatment of AD in order to call attention to this plant as a novel alternative in the treatment of Alzheimer's disease.

## 2. *Terminalia chebula* Retzius

*Terminalia chebula* Retzius (Family: Combretaceae), as a shade and ornamental tree with 250 species, is a medicinal plant that grows in the Middle East and tropical regions such as India, China, and Thailand. It can grow to be 25 meters tall and has a variable appearance and spreading branches. The color of the bark is dark brown and is usually cracked. Leaves are thin, elliptic-oblong, cordiform at the base, elliptical, and 7–12 cm long and 4–6.5 cm in width and have a leathery form with entire margins. The upper surface of the leaves is glabrous opposite of the surface beneath. The flowers are futile with a white to yellowish color and unsightly odor. Flowers have 5–7 cm long spikes, simple or branched, about 4 mm across. The ovary is inferior with 10 stamens. Fruits are yellow to orange-brown when ripe and 2.5–5 cm long and unruffled with an ovate-drupe shape [15, 61].

**Figure 1:**
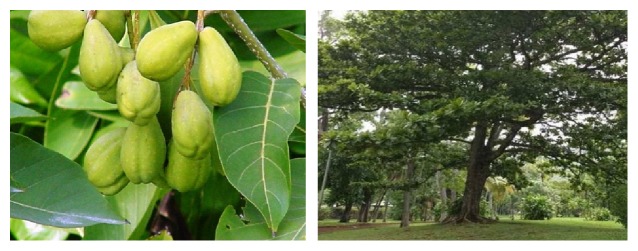
*T. chebula* tree and fruits.

Moreover, it is called by various names by the local people. For example, in the Thai language the plant's common name is “Kot Phung Pla,” and in Indian it is called “Kadukkaai”; and its other names are Black Myrobalan, Ink Tree, or Chebulic Myrobalan (Figure 1) [15].

### 2.1. Scientific Classification

Its classification is Kingdom: Plantae, Division: Magnoliophyta, Class: Magnoliopsida, Order: Myrtales, Family: Combretaceae, Genus:* Terminalia*, and Species:* Chebula* [15].

### 2.2. Phytochemical Compositions of* T. chebula*

#### 2.2.1. Hydrolysable Tannins

Tannins, as a part of the phenolic compounds, are oligomeric and have multiple structural units with free phenolic groups and their molecular weight ranges from 500 to 3000 D. The fruit pulp and dried pericarp of the seeds contain the highest amount of tannins [62]. Tannins consist of hydrolysable and nonhydrolysable tannins and hydrolysable tannins (i.e., gallotannins and ellagitannins) are the main compounds in* T. chebula*.

Gallotannins and ellagitannins are polymers found in the fruits of* T. chebula *[63]. Gallotannins contain gallic acid that has esterified and bonded with the hydroxyl group of a polyol carbohydrate such as glucose [64]. Ellagitannins are formed when oxidative linkage occurs in the galloyl groups in 1,2,3,4,6-pentagalloyl glucose. Ellagitannins differ from gallotannins in that their galloyl groups are linked through C–C bonds, whereas the galloyl groups in gallotannins are linked by depside bonds [65, 66].

Chebulagic acid, a benzopyran tannin, is widely distributed in several plant families: the Combretaceae [67], Euphorbiaceae, Leguminosae, Anacardiaceae, and Fabaceae [31]. Also, in the Combretaceae Family, chebulagic acid is the main constitute of the fruits* T. bellerica, T. chebula, *and* Emblica officinalis *[68].

Chebulinic acid, also known as 1,3,6-tri-*O*-galloyl-2,4-chebuloyl-*β*-D-glucopyranoside, is an ellagitannin found in the fruits of* T. chebula* or in the leaves of* T. macroptera* [31, 68].

#### 2.2.2. Phenolic Compounds

Phenolic compounds include ellagic acid, a natural potent phenolic antioxidant found in the fruits of* T. chebula* [13]; gallic acid, a trihydroxybenzoic acid found as a part of hydrolysable tannins [69]; tannic acid, a polymer of gallic acid molecules and glucose [70]; and chebulic acid, another phenolic compound in the ripe fruits of* T. chebula and* a component of transformed ellagitannins such as chebulagic acid or chebulinic acid [41].

Glycosides are other phenolic compounds that are widely present in the* Terminalia*, rhubarb,* Senna*, and* Aloe* species. The pericarp of the* T. chebula* fruit contains anthraquinone glycosides [71]. Table 1 shows the main ingredients of* T. chebula* and their chemical structures.

#### 2.2.3. Miscellaneous Compounds

In addition to the above, there are some other compounds that are also present and contribute towards the activity of the plant such as palmitic [72], stearic [73], oleic [74], linoleic [75], and arachidic acids [76], which are present in the fruit kernels.

#### 2.2.4. Methods of Search

This research included articles from 1960 to 2015 that were found in various electronic databases: PubMed, Science Direct, Scopus, Web of Science, Scirus, and Google Scholar by using the search words:* T. chebula*, AD, neuroprotection, medicinal plant, antioxidant, ellagitannin, gallotannin, gallic acid, chebulagic acid, and chebulinic acid. Only current articles that reported the effects of* T. chebula* on AD were included in our study. Then, a comparison of the related mechanisms and evaluation of pharmacology effects in the treatment and progression of AD was done.

### 2.3. *T. chebula* and Alzheimer's Disease

#### 2.3.1. Acetyl Cholinesterase (AChE)

One of the hypotheses about AD is acetylcholine (ACh) deficiency in the synaptic cleft of the cerebral cortex that causes memory disturbance. Acetyl cholinesterase, also known as AChE or acetyl-hydrolase, is a hydrolase that hydrolyzes the neurotransmitter acetylcholine. AChE is found at mainly neuromuscular junctions and cholinergic brain synapses where its activity serves to terminate synaptic transmission. It is the primary target of inhibition by organophosphorus compounds such as nerve agents and pesticides. AChE has a very high catalytic activity; each molecule of AChE degrades about 25000 molecules of ACh per second, approaching the limit allowed by diffusion of the substrate [77]. During neurotransmission, ACh is released from the nerve into the synaptic cleft and binds to ACh receptors on the postsynaptic membrane, relaying the signal from the nerve. AChE, also located on the postsynaptic membrane, terminates the signal transmission by hydrolyzing Ach [78]. AChE is found in many types of conducting tissue: central and peripheral tissues, motor and sensory fibers, nerve and muscle, and cholinergic and noncholinergic fibers. The activity of AChE is higher in motor neurons than in sensory neurons [79]. AChE is also found on the red blood cell membranes [80]. Traditionally, necrosis is the pathway that had been thought to cause cell death in AD; however, now it is thought that ACh containing neurons are the most important pathway of neuronal death in AD. The cell death mechanisms in AD are deposition of A*β*, reduced energy metabolism and/or mitochondrial dysfunction, excitotoxicity, and oxidative stress and free radical production. These four mechanisms contribute to necrosis in various diseases such as stroke and AD. Inflammatory responses and their effects on trophic factor function have also been proposed as a main factor for cell death in AD. AChE induces an apoptotic and necrotic cell death by inducing membrane depolarization and NMDA receptor activation with consequent Ca^2+^ influx and modulation of the *α*7 nicotinic acetylcholine receptor in hippocampal cultures. Therefore, AChE has a main role in neurodegeneration and AChEIs could be effective in neuroprotection [81–83]. Increasing expression of splice variants of AChE (R and S) as neuroprotective forms of AChE, with AChEIs, has been effective in preventing the progression of AD. In a study, expression of AChE-R and AChE-S decreased over a period of 12 months in untreated patients. In contrast, treatment with tacrine causes an upregulation in both variants (up to 117%) [84].

Inhibition of AChE leads to accumulation of ACh in the synaptic cleft and results in impeded neurotransmission [78]. The acetyl cholinesterase inhibitors (AChEIs) attenuate the cholinergic deficit underlying the cognitive and neuropsychiatric dysfunctions in patients with neurodegenerative disorders. So, inhibition of brain AChE has been the major therapeutic target of treatment strategies for AD, Parkinson's disease, senile dementia, ataxia, and myasthenia gravis [85]. There are a few synthetic medicines with this mechanism, for example, tacrine, donepezil, and the natural product-based rivastigmine, to treat cognitive dysfunction and memory loss associated with AD [86]. However, these compounds have been reported to have their adverse effects including gastrointestinal disturbances and problems associated with bioavailability, which necessitates the interest in finding better AChE inhibitors from natural sources [87].

In this section we discuss the effectiveness of* T. chebula* and its constituents on brain functions.

#### 2.3.2. Anticholinesterase Properties

Anticholinesterase properties of* T. chebula *have been reported in many studies [3, 88–95]. In Sancheti et al.'s study, the inhibitory effects of* T. chebula* on AChE and butyrylcholinesterase (BChE) were reported. In the in vitro study, methanolic crude extract of the fruits of* T. chebula *with a concentration of 5 mg/mL inhibited AChE and BChE about 89% and 95%, respectively. Sancheti et al. extracted 1,2,3,4,6-penta-O-galloyl-*β*-D-glucose (PGG) with a gallotannin structure from* T. chebula *by chromatographic methods, and they showed it to be the most potent AChE and BChE inhibitor. Bioassay of PGG exhibited its concentration-dependent inhibitory activity on AChE and BChE with IC_50_ values of 29.9 ± 0.3 *μ*M and 27.6 ± 0.2 *μ*M, respectively. Then, for deducing the inhibitory activity of PGG, a TLC assay was done with tacrine as a positive control, and the AChE inhibitory effect was visualized clearly in the assay [88]. In addition to the inhibitory effect of methanolic fruit extracts of* T. chebula* on AChE, the ethyl acetate fraction shows relatively remarkable AChE inhibitory activity. Sulaiman et al. showed that ethyl acetate fraction with doses of 1, 5, 15, and 25 mg/mL inhibited AChE by 29.36%, 32.44%, 45.82%, and 62.32%, respectively. This fraction was more potent than the chloroform and methanolic fraction in AChE inhibition [91]. In a similar in vitro study, Vinutha et al. used the methanolic and aqueous extracts of the fruit of* T. chebula* and the data showed that IC_50_ values of the aqueous extract of* T. chebula* (minimum inhibition: 12.45%) are more potent than the methanolic extract (minimum inhibition: 1.21%) [3].

In addition to the effects of gallotannins on AChE inhibition, tannic acid as an active component of* T. chebula* has a potential inhibition effect on AChE. Upadhyay and Singh showed the competitive inhibition of AChE by tannic acid in vivo and in vitro. This study focused on the effects of tannic acid from the* T. chebula* fruit. In vivo treatment with 80% of LC_50_ of tannic acid during 96 h caused a remarkable inhibition in AChE activity in the nervous tissue of* Lymnaea acuminate*. Furthermore, maximum inhibition of AChE activity was detected when the tissue was exposed to 80% of LC_50_ of tannic acid for 96 h, so inhibition of AChE was time- and dose-dependent in the 96 h process. In vitro treatment showed that tannic acid caused dose-dependent AChE inhibition significantly, such that 0.04 mM of tannic acid reduced the AChE activity to 37% of the control in the nervous tissue of* L. acuminata* [92]. In addition, another in vivo research on AChE activity showed that tannic acid completely neutralized the AChE activity in* Naja kaouthia* venom [96].

Parle and Vasudevan in their in vivo study showed that* Abana *(a mixture of medicinal plants containing* T. chebula*) with doses of 50, 100, and 200 mg/kg administered orally for 15 days can reduce the brain AChE activity in young and aged mice. Passive avoidance apparatus and maze tests were performed on day 16 and the results showed a remarkable dose-dependent reduction in transfer latency and a significant increase in step-down latency tests, indicating significant improvement in memory [93].

Another similar study was done by Dhivya et al. In this in vitro study, AChE inhibitory activity of various plant species containing* T. chebula* was reported to have AChE inhibition of 41.06 ± 5.6% (0.1 mg/mL) as well [95].

In a similar study, AChE inhibition of the methanolic extract of* T. chebula* showed a moderate inhibition with an extract with IC_50_ value of 180 ± 14.6 *μ*g/mL and the percentage of AChE inhibition was 41.06 ± 5.6 at 0.1 mg/mL [97]. In addition, the percentage of AChE inhibition in the extract of* T. chebula *was 13% (IC_50_ not detected) [14]. Furthermore, in another research,* T. chebula* could inhibit 89% of AChE activity (5 mg/mL) and 95% of BChE activity (5 mg/mL) [88].

Gallic acid and ellagic acid, as phenolic compounds in the fruits of* T. chebula*, could also inhibit AChE. Nag and De in their study showed that the inhibitory effect of the methanolic extract of* T. chebula* (gallic acid: 0.25 *μ*g, ellagic acid: 0.08 *μ*g) has a linear relationship with increasing dosage (IC_50_: 10.96) [89].

#### 2.3.3. Anti-Inflammatory Properties

Cyclooxygenase (COX) and 5-lipoxygenase (5-LOX) are the key enzymes that are involved in inflammation. Reddy et al. investigated chebulagic acid, as a COX-LOX dual inhibitor, from the ethanolic extract of fruits of* T. chebula*. The results showed that chebulagic acid has a potent COX-LOX dual inhibition activity with IC_50_ values of 15 ± 0.288, 0.92 ± 0.011, and 2.1 ± 0.057 *μ*M for COX-1, COX-2, and 5-LOX, respectively [76].

The water soluble fraction of* T. chebula* (WFTC) was effective against systemic and local anaphylaxis. Shin et al. showed that the injection of WFTC with doses of 0.01–1.0 g/kg inhibited anaphylactic shock 100%. When WFTC was pretreated at concentrations ranging from 0.005 to 1.0 g/kg, the serum histamine levels were reduced dose-dependently. In addition, this study showed that WFTC increased anti-dinitrophenol and IgE-induced tumor necrosis factor- (TNF-) *α* production [98].

Lee et al. in their study showed that gallic acid (25 *μ*g) suppressed nuclear factor kappa B (NF-*κ*B) activity and cytokine release. It also remarkably reduced the cyclic AMP response element binding protein/p300 (CBP/p300, a NF-*κ*B coactivator) gene expression, acetylation levels, and CBP/p300 histone acetyltransferase activity. Therefore, gallic acid derived from the extracts of several plants especially* T. chebula* was shown to exert anti-inflammatory activity via the downregulation of the NF-*κ*B pathway in the development of inflammatory diseases, both in vitro and in vivo [30, 99–101].

Kim et al. showed that the inhibition of NF-*κ*B acetylation with gallic acid resulted in reduced cytokine production in microglia cells and the protection of neuronal cells from amyloid *β*- (A*β*-) induced neurotoxicity. In addition, this research showed a restorative effect of gallic acid on A*β*-induced cognitive dysfunction in mice in Y-maze and passive avoidance tests. Therefore, gallic acid has several roles in neuroinflammatory diseases including (1) preventing A*β*-induced neuronal cell death by inhibiting RelA (as a gene encoding NF-*κ*B) acetylation and cytokine production; (2) protecting neuronal cells from primary microglia-mediated A*β* neurotoxicity; (3) restoring A*β*-induced memory deficits in mice; and (4) suppressing in vivo cytokine production by inhibiting RelA acetylation in brain [101]. Another similar study showed that polyphenolic extracts (containing gallic acid) of medicinal plants such as* T. chebula* are able to inhibit A*β* aggregation, reduce A*β* production, and protect against A*β* neurotoxicity, in vitro [102]. Kim et al. reported the inhibitory effects of gallic acid on inflammatory responses via NF- *κ*B and p38 mitogen-activated protein kinase pathways [103].

In another study, chebulagic acid showed a potent anti-inflammatory effect in lipopolysaccharide- (LPS) stimulated RAW 264.7 (mouse macrophage cell line). Reddy and Reddanna showed the effectiveness of chebulagic acid against inflammatory diseases by different mechanisms such as inhibition of nitric oxide (NO) and prostaglandin E_2_ production, downregulation of iNOS (inducible nitric oxide synthases), COX-2, 5-LOX, TNF-*α*, interleukin 6, and inhibitory effects on NF-*κ*B, and decreases in nuclear p50 and p65 protein levels. In addition, the generation of reactive oxygen species (ROS) and phosphorylation of p38, ERK 1/2, and JNK in LPS-stimulated RAW 264.7 cells was suppressed by chebulagic acid in a concentration-dependent manner [44]. In another research, chebulagic acid from the immature seeds of* T. chebula* significantly suppressed the onset and progression of collagen induced arthritis in mice [104]. In a similar study, the aqueous extract of the dried fruit of* T. chebula* showed an anti-inflammatory effect by inhibiting iNOS [105]. Patil et al. in their study showed that administrating the fruit of* T. chebula* (500 mg/kg) via inhibition of iNOS expression can be significantly effective in the progression of inflammation [106]. In vivo anti-inflammatory activity of* T. chebula* fruit extract at different doses (range: 50 to 500 mg/kg) was evaluated against carrageenan-induced inflammation in rats. In this study, Bag et al. reported that 250 mg/kg (administrated orally) caused a 69.96% reduction in carrageenan-induced rat paw edema. This finding suggests that free radical quenching may be one of the mechanisms for its anti-inflammatory activity [107]. Triphala, an Indian Ayurvedic herbal formulation containing* T. chebula*, at a dosage of 1 g/kg, showed an anti-inflammatory effect. Sabina and Rasool in their research showed that the mechanisms of anti-inflammatory properties of this plant include (a) inhibition of lysosomal enzyme release, (b) a significant decrease of inflammatory mediator TNF-*α*, and (c) a decrease of beta-glucuronidase and lactate dehydrogenase enzymes release. The anti-inflammatory effect of Triphala might be due to the presence of phenolic components and flavonoids [108]. In another study, Ramani and Pradhan showed that the acetone extract of* T. chebula* fruits had a significant effect on controlling Complete Freund's Adjuvant-induced arthritis. This effect leads to a good reduction in paw edema and joint thickness (reduction of ESR values and RF values) in comparison with dexamethasone [109].

#### 2.3.4. Antioxidant Properties

Decreases in antioxidant defense or increases in oxidant status in the body tend to lead to the condition called oxidative stress, which plays a major role in neurological degeneration such as AD [110]. Reactive oxygen species and oxidative stress increase the formation of amyloid-*β* and senile plaques in the brain are the hallmark of AD [111]. High lipid content, deficient antioxidant values, and high oxygen consumption in the brain are the reasons the central nervous system is vulnerable to oxidative stress. Increased lipid peroxidation, decreased glutathione levels, increased dopamine turnover, and elevated iron and aluminum levels in the brain support the role of oxidative stress in Parkinson's, Amyotrophic Lateral Sclerosis, and AD [112].

In Table 2, we have summarized the roles of oxidative stress in the pathogenesis and progression of AD. Thus, reducing oxidative stress by enhancing antioxidant defense or decreasing the production of reactive oxygen species may be effective in treating AD.

Antioxidant effects of* T. chebula* extract (100 *μ*g/mL) were compared with reference radical scavengers such as quercetin, gallic acid, and t-butylhydroquinone, and* T. chebula* extract showed 95% activity with IC_50_ 2.2 *μ*g/mL [14].* Terminalia* species such as* T. chebula* and* T. arjuna* with a high content of phenolic constituents showed strong antioxidant and antiaging properties [88]. Oral administration of aqueous extracts of* T. arjuna* causes significant elevation in the activities of antioxidant enzymes such as superoxide dismutase, catalase, and glutathione S-transferase. The strong antioxidant action of the aqueous extract of the* Terminalia* species may play a role in treating age-related diseases such as AD [127]. A similar study done by Mathew and Subramanian showed that the antioxidant activity of the methanolic extract of* T. chebula* (0.1 mg/mL) and IC_50_ was 86.3% and 0.5 *μ*g/mL, respectively. In this study the highest amount of antioxidant activity among 20 plants was found in* T. chebula* due to the presence of gallic acid and ellagic acid. All 20 plants used in this study were traditional neuroprotective plants that have been used for many years and among them* T. chebula* was the most potent antioxidant and moderate inhibition of AchE [97]. In the evaluation of antioxidant, anti-inflammatory, membrane-stabilizing, and antilipid peroxidative effects of the hydroalcoholic extract of* T. chebula* fruits in arthritic disorders, Bag et al. showed that the fruit extract of* T. chebula* (10 to 100 *μ*g/mL) in carrageenan-induced inflammation in rats reduced the formation of thiobarbituric acid reactive substances in the liver with IC_50_ 94.96 mg/kg. The IC_50_ of the extract in DPPH radical-scavenging activity was 42.14 *μ*g/mL [107]. Antilipid peroxidative capacity of* T. chebula* was attributed to higher phenolic content, reducing ability, Fe(II) chelating ability, and free radical-scavenging activity. In Khan et al.'s study, the aqueous extracts of yellow* T. chebula* and black* T. chebula* showed IC_50_ values of 20.24 ± 0.9 *μ*g/mL and 17.33 ± 1.1 *μ*g/mL in the scavenging of the DPPH radical in mice brains, respectively [128]. In an in vitro study done to investigate the biological activities of phenolic compounds and triterpenoid constituents of* T. chebula*, 9 phenolic compounds and 8 triterpenoids were extracted. Radical-scavenging activities of the phenolic compounds were higher than the triterpenoids compounds and had potent inhibitory activities against melanogenesis at a concentration of 10 *μ*M and the IC_50_ was 1.4–10.9 *μ*M. These results showed that* T. chebula* with a high content of polyphenolic ingredients was a potent antioxidant [16]. The extract of the dried fruit pulp of* T. chebula* (600 mg/kg) could diminish acetic acid-induced colitis in rats with antioxidant effects (free radical-scavenging, antioxidant enzymes enhancing activity and decreasing lipid peroxidation) [129].

In the evaluation of neuroprotective effects, the inhibition of H_2_O_2_-induced PC12 cell death by* T. chebula* was investigated. H_2_O_2_ at 40 *μ*M for 12 h decreased cell viability to 59%. Also, all of the methanol, water, and 95% ethanol extracts of* T. chebula* increased cell viability at 0.5–5.0 *μ*g/mL, dose-dependently; and among them the water extract was more potent. Antioxidant effects of the extracts were compared and the results showed that methanol extract had good antioxidant activity based on the luminol-H_2_O_2_-horseradish peroxidase assay and the water extract was shown to have good antioxidant activities in cupric sulfate phenanthroline-H_2_O_2_ and luminol-H_2_O_2_ assays. Also, 95% ethanol extract showed good antioxidant activity in the pyrogallol-luminol assay [130]. Effects of* T. chebula* seed extracts on brain-derived neurotrophic factor (BDNF), Cu, Zn-superoxide dismutase (SOD1), and Mn-superoxide dismutase (SOD2) were evaluated by Park et al. Extract with a dose of 100 mg/kg once a day was administrated to gerbils for 7 days before induction of transient cerebral ischemia and the neuroprotective effect of the extract was evaluated in the CA1 hippocampal region on the fourth day after ischemia-reperfusion induction. The results showed that astrocytes and microglia remarkably decreased in the ischemic group compared with the vehicle-treated group and neuron nucleus (as the positive neuron) was distinctively abundant (62%) compared to the vehicle-treated ischemia group (62% versus 12.2%). Protein levels and activity of SOD1, SOD2, and BDNF were much higher in the treated group. These results confirmed the neuroprotective effects of* T. chebula* extracts against neuronal damage in hippocampus [131]. Recently, we have found that the alcoholic extract of* T. chebula* fruits significantly protects against quinolinic acid-induced neurotoxicity by alleviating the oxidative stress parameters (unpublished data).

Many studies used* T. chebula* to modulate the oxidative stress adverse effects in various models of oxidation damage and aging [132–134].* T. chebula* could enhance the antioxidant defense and modulate oxidative stress due to aging in the liver and kidney of aged rats compared to young albino rats [133] and could protect hepatocytes from damage due to oxidative stress and enhanced total thiol content [41]. Many studies have shown the antioxidant activity of* T. chebula* in various in vivo and in vitro models [16, 135, 136]. In addition, most of the researchers agree with this hypothesis that “pharmacophore, which contains large numbers of –OH group (such as polyphenols), has greater anti-aging properties than those with less numbers of –OH groups.” Therefore, two constituents that are potent phenolic compounds are ellagic acid and 4-O-xyloside of ellagic acid. These active ingredients were investigated for their activities on free radical-scavenging, elastase inhibition, expression of matrix metalloproteinase-1 (MMP-1), and type I collagen synthesis in normal human fibroblast cells. Thus, ellagic acid may be useful for the geriatric population [137, 138].

According to the points mentioned above regarding the antioxidant activity of* T. chebula* and the important role of oxidative stress in the pathogenesis of AD, we can conclude that the use of this plant may be effective against the progression of AD.

## 3. Conclusion

**Figure 2:**
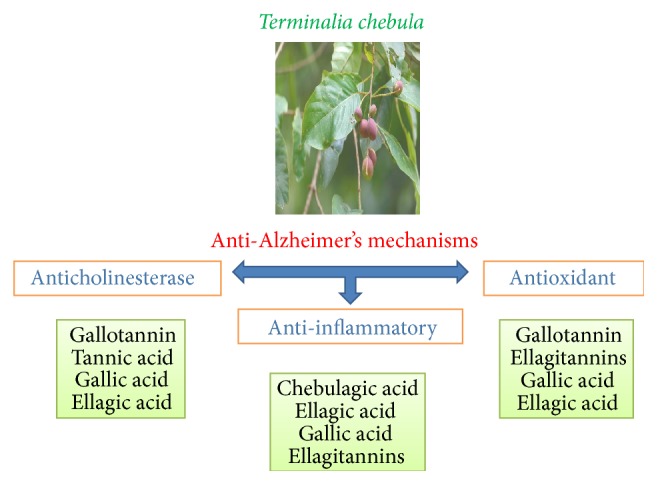
Anticholinesterase, anti-inflammatory, and antioxidant properties of* T. chebula* relevant to anti-Alzheimer's therapy.

Alzheimer's disease is a debilitating dementia, and only a limited number of therapeutic options are currently available to modify the manifestations of the disease.* T. chebula* has pharmacological activities relevant to dementia therapy (Figure 2). Different extracts from* T. chebula* have exhibited concentration-dependent inhibitory activities on AChE and BChE. Such AChE activities are also described for its active ingredients such as PGG, gallic acid, ellagic acid, and tannic acid. Anti-inflammatory properties of* T. chebula* have been well documented in different experimental systems that could be attributed to chebulagic or gallic acid.* T. chebula* with a high content of phenolic constituents exhibits strong antioxidant and neuroprotective properties in vitro and in vivo. The efficacy of* T. chebula* in treating AD should be compared with the current standard pharmacological treatment in animal and clinical testing and researches. Such studies should include the identification of the active principle(s) in order to improve the validation of the clinical trials. Until then, this review provides some evidences on the benefits of* T. chebula* in the treatment of Alzheimer's disease.

## Figures and Tables

**Table 1:** Structure and pharmacological properties of *T*. *chebula* active ingredients.

Compound	Category	Chemical structure	Pharmacological properties
Gallotannins	Hydrolysable tannin	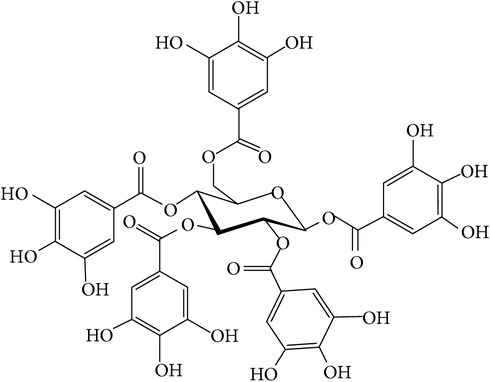	Antimicrobial [26], antioxidant [27]

Ellagitannins	Hydrolysable tannin	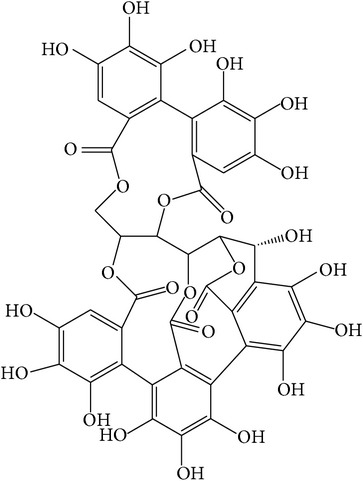	Anti-inflammatory, anticancer, cardiovascular protection [28], antioxidant, chemopreventive, antiapoptotic, anti-hepatocellular carcinoma (Anti-HCC) [29]

Gallic acid	Phenolic compound	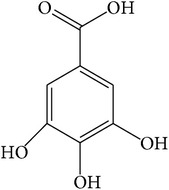	Anti-inflammatory [30], antimutagenic [31], cardioprotective [32], antioxidant [33], anticancer [34], antimicrobial [35], neuroprotective [36], immunosuppressive [37], improved cognition [38]

Chebulic acid	Phenolic compound	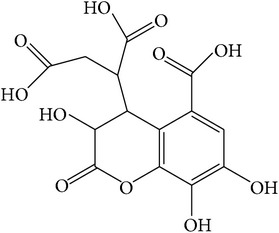	Anti-HCV [39], antidiabetic [40], hepatoprotective [41], immunosuppressive [37]

Chebulagic acid	Hydrolysable tannin	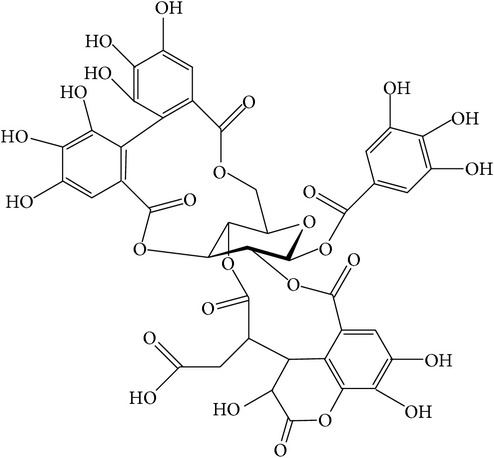	Hepatoprotective [41], antiviral [42], immunosuppressive [43], antidiabetic [44, 45], neuroprotective [46], antiangiogenesis [47], antiproliferative [48], anti-inflammatory [44]

Chebulinic acid	Hydrolysable tannin	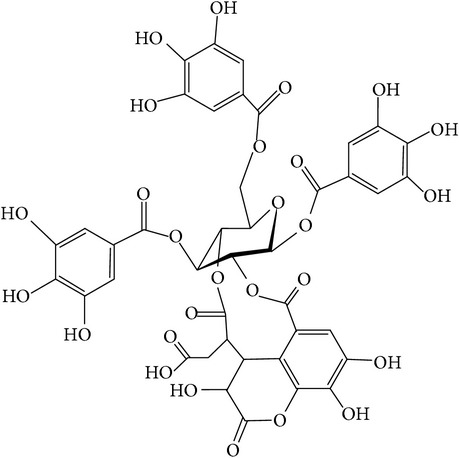	Antisecretory, cytoprotective [49], antiangiogenesis [50], antitumor [51]

Ellagic acid	Phenolic compound	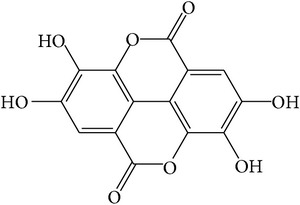	Antioxidant [52], anti-inflammatory [53], anti-diabetes-induced sexual dysfunction [54], hepatoprotective [55], antiarrhythmic [56], cognitive enhancer [57, 58]

Anthraquinone glycosides	Phenolic compound	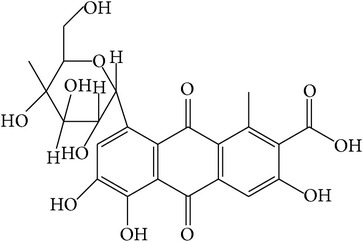	Neuroprotective [59], antidiabetic [60]

**Table 2:** Major effects of oxidative stress in the pathogenesis of Alzheimer's disease.

Effects of ROS	Result	Biological evidence(s)
Protein oxidation	Increased protein carbonyl content	Increased protein oxidation in frontal pole and occipital pole [113] Decreased ratio of (MAL-6)/(W/S) in AD hippocampus and inferior parietal lobule, decreased the W/S ratio in in vitro models of human synaptosomes oxidation by ROS [114]

DNA oxidation	Direct damage to DNA structure	3-fold increase in mitochondrial DNA oxidation in parietal cortex in AD [115] Increase in oxidative damage to nuclear DNA in AD compared with age-matched control subjects [116] 8-hydroxy-2-deoxyguanosine as a marker of DNA oxidation increases in AD [117]

Lipid peroxidation	Brain phospholipid damage	Increased TBARS levels in AD in hippocampus, piriform cortex, and amygdala [118] Increased lipid peroxidation of AD brain homogenates in vitro due to Fe-H_2_O_2 _ [119] Increased apoptosis in cultured DS and AD neurons inhibited by antioxidant enzymes [112, 120] Decrease in PC, PE, phospholipid precursors, choline, and ethanolamine in hippocampus and inferior parietal lobule in AD [121] Increased aldehydes as a cytotoxic agent in the brain of AD patients [122]

Antioxidant enzymes	Changes in enzymes contents	Elevated GSH-Px, GSSG-R, and CAT activity in hippocampus and amygdala in AD [118] Many studies showed no elevation in enzyme activity [123, 124] or decrease in activity [125]

AGE formation	Pathological changes in protein structure and action	Accelerates aggregation of soluble nonfibrillar A*β* and tau [126]

MAL-6: weakly immobilized protein bound spin label; W/S: strongly immobilized protein bound spin label; TBARS: thiobarbituric acid reactive substances; DS: Down syndrome; PC: phosphatidylcholine; PE: phosphatidylethanolamine; GSH-Px: glutathione peroxidase; GSSG-R: glutathione reductase; CAT: catalase; AGE: advanced glycation end products.
